# What is radiation-induced acute myeloid leukaemia/can it be accurately identified?

**DOI:** 10.1038/s41375-025-02561-2

**Published:** 2025-03-27

**Authors:** Christophe Badie, Robert Peter Gale

**Affiliations:** 1 Radiation Effects Department, Radiation, Chemicals, Climate and Environmental Hazards Directorate, UK Health Security Agency, Harwell campus, Chilton, Didcot, Oxfordshire OX11 0RQ UK; 2 Centre for Haematology, Imperial College of Science, Technology and Medicine, London, UK

**Keywords:** Health sciences, Cancer prevention

Exposure to ionizing radiations can cause and/or contribute to developing acute myeloid leukaemia AML; reviewed in [1]. How it does so is less certain. Most people believe the direct cause is physical damage to DNA resulting in a *driver* mutation(s) and that this is *necessary and* perhaps *sufficient* to cause AML. But is this so? Are their convincing experimental or clinical data supporting this mechanism? Might radiation simply act on someone predisposed to get AML by promoting or accelerating the process? Importantly, from a clinical perspective is there a mutational signature or biomarker allowing us to accurately identify someone with radiation-related AML? If so, should such persons receive more intensive therapy because of an adverse prognosis? We tackle these issues in our Perspective; some answers will surprise and likely upset readers of *LEUKEMIA*.

The terms *therapy-related AML, radiation-related AML* and *secondary* AML (implying an prior cause) are often used inter-changeably to describe leukaemia developing in someone exposed to ionizing radiations, usually in the context of radiation therapy. However, often details including age at exposure, latency, dose, dose-rate, field, fractionation, source-term parameters are unknown or disregarded. Also, there are often concomitant exposure(s) to anti-cancer drugs known to cause or contribute to developing leukaemia making the contribution of radiation exposure, if any, less certain.

The 2022 5th edition of the World Health Organization Classification of Haematolymphoid [*sic*] Tumours: Myeloid and Histiocytic/Dendritic Neoplasms recommends a category of *secondary* myeloid neoplasms after cytotoxic therapy in persons with prior exposure to large-field radiation therapy for an unrelated neoplasm [2]. For reasons we discuss above we disagree with this designation, especially as *secondary* implies an unproved aetiological link. Therapy-related AML is no longer a entity in the 2022 International Consensus Classification but a qualifier attached to a diagnosis of AML [3]. Again, we disagree. Both classifications wander into the realm of speculation.

Ionizing radiations, radiations with sufficient energy to break an electron away from an atom, are mutagens and carcinogens resulting in mutations with a characteristic pattern [4]. Exposure of human cells of different origins to 1 Gy (absorbed dose) results in an average of 2.33 mutations *per* Gb DNA, mostly indels (insertion and/or deletions) [5]. Ionizing radiations can be a cancer *initiator, promoter* or *accelerator* (see below) depending on several factors such as latency to diagnosis, radiation quality, dose, dose-rate, route of exposure, sensitivity of individual cells in the target tissue/organ, tissue kinetics and cell organisation. Other factors may also influence the type and risk of radiation-related leukaemia such as sex, age at exposure and hormonal milieu [6].

The main obstacle in understanding the mechanism(s) of radiation-induced cancers is identifying the initiating event, usually thought to be a somatic DNA modification such as a chromosome aberration or smaller mutation in a target cell. Importantly, having a mutation able to cause or contribute to a cancer does not guarantee a cancer will develop within someone’s lifetime. Although, there is little doubt the initial event is physical damage in DNA there are few direct experimental confirmations specific cancer-initiating mutations or aberrations are the dominant, rate-limiting step in radiation-induced cancers [7].

There are no genetic alterations specific to radiation-induced cancers including AML [8]. Finding such a biomarker following clonal expansion and cancer development and diagnosis is further complicated because radiation-induced cancers are histologically, cytogenetically and molecularly indistinguishable from cancers seemingly occurring spontaneously. Consequently, it is unclear how to accurately identify people with AML caused by or contributed to by radiation exposure [9].

Studies in animals can potentially improve our understanding of mechanisms underlying radiation-induced AML. In CBA/H mice exposure to 3 Gy X-ray whole body radiation exposure results in a 15 percent incidence of AML after an average 15-month latency; the background incidence is negligible. Interstitial deletions of chromosome 2 always including *Spi1* (*PU1* in humans), a haematopoietic transcription factor, are detected in bone marrow cells soon after exposure and considered to be the 1^st^ event in a 2-hit model of radiation-induced leukaemogenesis [10]. Point mutations in the DNA-binding domain (Spi1^R235C^) in the trans allele are often the 2^nd^ event [11]. 3 minor pathways include internal tandem duplication (Flt3-ITD) [12], *Kras* mutations and *Spi1* promoter methylation [13].

In CBA mice, a Spi1^R235C^ mutation confers hyper-sensitivity to radiation-induced AML resulting in 100 percent penetrance [14]. Remarkably, and unexpectedly, all control mice also develop AML by 12 months of age separated only in time from irradiated mice who developed it on average with a 7-month latency. In this model it appears radiation exposure *accelerates* AML development rather than initiating it as the driving mutations (interstitial deletion of chromosome 2 including Spi1 and Spi1^R235C^ point mutation in the trans allele) are similar. Whether *acceleration* rather than *initiation* operates in humans is unknown.

It is important to mention how the terms *promotion* and *acceleration* are used in different disciplines. In studies of chemical carcinogenesis *promotion* is a *necessary* step for leukaemia to develop; no *promotion*, no leukaemia. In contrast, in radiobiology *promotion* and *acceleration* are synonymous. In humans exposed to radiation the term *acceleration* after radiation exposure means people who would develop leukaemia anyway (if they live sufficiently long) develop leukaemia earlier and/or are diagnosed earlier than they would have had without being exposed to radiation. We use the term *promotion* as a necessary step to leukaemia development and *acceleration* as decreasing the interval to when leukaemia is diagnosed. Promotion is *necessary but insufficient* for leukaemia development whereas *acceleration* is neither *necessary nor sufficient*.

Although radiation-induced leukemogenesis is attributable to direct DNA damage, the potential role of cell fitness, competition for the bone marrow *niche* and/or other factors or combinations thereof is controversial [15]. Mice with Trp53 mutations are also extremely susceptible to radiation-induced cancers [16]. Cells with *driver mutations* in Trp53 outcompete wild-type cells following exposure to low-dose radiation [17].

Contrary to popular opinion most data suggest mutations alone are insufficient for cancer development with *promotion* rather than *initiation* the rate-limiting step [18]. Wong et al. reported selection of pre-existing haematopoietic stem cell (HSC) clones with mutations in *TP53* following anti-cancer therapy [19]. This pattern is consistent with the frequencies of *TP53* mutations in 5–10 percent of people with de novo myelodysplastic neoplasms (MDS*) versus* 30–40 percent of those with MDS or AML proposed to be therapy-related [20]. Damage to the bone marrow micro-environment from radiation can cause inflammation and/or immune suppression hence promoting AML development. However, most data suggest no increased risk of AML in people with immune suppression [21].

Whole genome sequencing is being used to define mutation *signatures* which might explain what underlies cancer development. People with AML have one of the fewest mutations *per* Gb DNA compared with other cancers (8 per Gb) [22]. Behjati and colleagues reported an excess of small deletions and balanced chromosome inversions in sarcomas thought to be caused by radiation [23]. These deletions show no density variation across the genome or correlation with sequence context. Somatic mutation signatures from radiation exposure are reported in mouse HSCs the most common of which is a significant increase in non-repeat deletions without micro-homology generated by the DNA double strand break repair by non-homologous end joining (NHEJ) [24]. In this model many HSCs originate from a HSC surviving radiation with varying degrees of clonal expansion and dynamics correlated with radiation dose and fractionation. Dominance of a clonal cell population derived from one HSC might reflect a population drift accelerated by radiation exposure rather than selective advantages of certain HSCs from pre-existing somatic mutations. Whether this is a universal radiation-induced mutation signature is unknown. Notably, radiation-related AML has a relatively brief latency suggesting a relatively simple mechanism of leukaemogenesis compatible with *promotion* or *acceleration* rather than with *initiation* [25].

Nakamura hypothesized the increased risks of acute lymphoblastic (ALL) and chronic myeloid leukaemia (CML) In studies of the A-bomb survivors is almost entirely attributable to a few persons with predisposing translocations. Whether a similar mechanism might operate in *young-at-exposure* cases of radiation-related AML in the A-bomb survivors and after radiation therapy is unknown [26].

Clonal haematopoiesis may also be a risk factor for developing radiation-related AML. In 2016, an estimated 3 million Americans with cancer received radiation therapy, a number expected to increase in the next several years [27]. By 2050, the global population of people aged > 60 years will double to 2.1 billion and numbers of persons > 80 years or older is expected to reach 426 million [28]. These are large populations with clonal haematopoiesis potentially at-risk for radiation therapy induced-AML.

In conclusion, data from epidemiological studies and mouse models indicate radiation-exposure can cause, promote and/or accelerate development of AML. Because there is no mutation *signature* or biomarker specific to radiation-related AML designating someone as having radiation-related AML is probabilistic, not deterministic and physicians should understand the uncertainty inherent in this designation. Mis-attribution of causality can have adverse or even fatal consequences based on a perceived high-risk of therapy-failure. We suggest therapy-decisions such as a haematopoietic cell transplant should not be made based on assuming someone has radiation-related AML.

Future studies may identify a mutation *signature* of radiation-induced AML providing a bridge from epidemiological findings to insights into the earliest events. However, finding such a signature is far from certain; we think it unlikely. So, reverting to our title: *What Exactly is Radiation-Induced Acute Myeloid Leukemia/Can it Be Accurately Identified* we suggest the mechanism in a mouse model but not presently confirmed in humans. Presently, radiation-induced AML in humans cannot be accurately identified and the term should not be used without stating the associated uncertainty.

## Disclaimer

CB: This is my view and does not imply agreement of the UK Health Security Agency.
